# Low-level laser therapy effectiveness in reducing initial orthodontic archwire placement pain in premolars extraction cases: a single-blind, placebo-controlled, randomized clinical trial

**DOI:** 10.1186/s12903-020-01191-7

**Published:** 2020-07-20

**Authors:** Mohammad Moaffak A. AlSayed Hasan, Kinda Sultan, Mowaffak Ajaj, Iva Voborná, Omar Hamadah

**Affiliations:** 1 Department of Orthodontics and Dentofacial Orthopaedics, Faculty of Dental Medicine, Damascus University, Damascus, Syria; 2 Department of Prosthodontics, Institute of Dentistry and Oral Sciences, Palacký University in Olomouc, Olomouc, Czech Republic; 3 Department of Oral Medicine, Faculty of Dental Medicine, Damascus University, Damascus, Syria; 4 Higher Institute for Laser Researches and Applications, Damascus University, Damascus, Syria

**Keywords:** Low-level laser therapy, Orthodontic pain, Initial orthodontic archwire placement, Spontaneous and chewing pain

## Abstract

**Background:**

The objective of this randomized clinical trial was to evaluate Low-Level Laser Therapy (LLLT) effectiveness in spontaneous and chewing pain reduction following initial orthodontic archwire placement.

**Methods:**

26 patients (mean age 20.07 ± 3.13 years) with maxillary Little’s Irregularity Index (LII) of 7 mm or more that indicates first maxillary premolars extraction and no medications intake were eligible for this trial. Patients were randomly assigned with 1:1 ratio using simple randomization technique to receive either LLL or placebo treatment. Blinding was applicable for patients only. In the laser group, patients received a single LLL dose (wavelength 830 nm, energy 2 J/point) in four points (2 buccal, 2 palatal) for each maxillary anterior tooth root. Patients in the placebo group had the same laser application procedure without emitting the laser beam. Patients were asked to score spontaneous and chewing pain intensity by filling out a questionnaire with a 100-mm Visual Analogue Scale (VAS) after 1, 6, 24, 48, and 72 h of treatment application. Independent t-test was used to compare the mean pain scores between the laser and placebo groups for both spontaneous and chewing pain at each studied time point.

**Results:**

No dropout occurred so the results of the 26 patients were statistically analyzed. Despite some clinical differences observed between the two groups, no statistical significance was found for each studied time point (*p* > 0.05) for both spontaneous and chewing pain except after 72 h for chewing pain with a VAS score of (18.84 ± 13.44) mm for the laser group compared to (38.15 ± 27.06) mm for the placebo group.

**Conclusions:**

LLLT, with the suggested parameters, is not effective in pain reduction following initial orthodontic archwire placement.

**Trial registration:**

**Name of the registry:**Clinicaltrials.gov

**Trial registration number:**NCT02568436.

**Date of registration:** 26 September 2015 ‘Retrospectively registered’.

## Background

Pain is unpleasant sensation caused by various degrees of nerve endings irritation. Despite the differences in pain perception among people, it is still measurable and has a great role in patients’ decision about completing their orthodontic treatment [1]. In orthodontics, leveling and alignment stage involves inserting archwires in a specific sequence in order to get teeth aligned [2]. Inserting orthodontic archwires - especially the initial archwire - is accompanied with generating orthodontic forces that lead to reactions in the periodontal tissues like edema and acute ischemia. These inflammatory reactions induce the secretion of inflammatory mediators that make the orthodontic treatment a painful procedure [3, 4]. According to studies 95% of orthodontic patients feel pain during orthodontic treatment, while 8–30% of them halt their treatment due to pain [5]. Beside, 50% of orthodontic patients suffer moderate to severe difficulties in daily activities like chewing foods [4]. Orthodontic pain usually begins several hours after orthodontic force application and reaches its peak after 18–36 h, then diminishes gradually till it disappears after 7 days [6]. Many methods have been studied in order to reduce orthodontic pain. These methods included the behavioral cognitive therapy [7], chewing gum or hard or soft bite wavers [8], using medications like Ibuprofen or topical application of anesthetic gel [9], and the application of vibrational forces [10] or Low-Level Laser Therapy (LLLT) [11]. The most commonly used method is taking medications especially Non-Steroidal Anti-Inflammatory Drugs (NSAIDs) [12]. The analgesic effects of these medications are attributed to their ability to inhibit Prostaglandin secretion in the injury site. However, they have some side effects like bleeding disorders, ulcers, and reducing the orthodontic tooth movement rate [12, 13].

LLLT has been recently applied as a nonpharmacological method to reduce orthodontic pain because of its analgesic and anti-inflammatory effects attributed to increased blood flow due to alleviating Prostaglandin levels and inhibiting COX-2 enzyme secretion [14, 15]. It has low energy that is enough so that it does not raise the temperature of the targeted tissues above 36.5^o^ or more than the body temperature [16]. This treatment method is considered a simple, noninvasive method that can be used in dental practice for many purposes like orthodontic tooth movement acceleration and pain reduction [17–19].

Many studies have evaluated the efficiency of LLLT in alleviating orthodontic pain. Those studies used different protocols (application points, application time) and laser parameters (energy, wavelength, power, energy density) of LLLT and evaluated pain perception in various orthodontic treatment stages (elastomeric separators placement [11, 14], first orthodontic archwire placement [8, 20], canine retraction [17, 21, 22], final orthodontic archwire placement [1]). Accordingly, the results varied between emphasizing LLLT efficiency in pain reduction and refusing it. Few previous studies evaluated pain after initial orthodontic archwire placement [8, 20, 23–25]. Although they all found laser effective in several degrees, yet the evidence is not clear according to systematic reviews [12, 18] because of the high or unclear risk of bias or the incomplete design of some of these studies that make the results indecisive. Beside, no previous study evaluated pain reduction with severe crowding that indicates first premolars extraction. Therefore, there is still a need for more randomized controlled trials to evaluate laser efficiency.

The aim in this study is to evaluate LLLT effectiveness in spontaneous and chewing pain reduction following initial orthodontic archwire placement in severe crowding cases that indicates premolars extraction.

The null hypothesis is that LLLT is not effective in reducing orthodontic pain following initial orthodontic archwire placement.

## Methods

This study was a 2-arm parallel-group single-center single-blind non-inferiority placebo-controlled randomized clinical trial (RCT) with allocation ratio of 1:1. The CONSORT statement was used as a guide to write this article [26]. This RCT was registered in the clinicaltrials.gov database with identifier number (NCT02568436). No changes to the methods occurred after trial commencement.

Orthodontic patients in need for fixed orthodontic appliance treatment to decrowd severe maxillary anterior teeth crowding were enrolled in this study by the first author from patients attending the Department of Orthodontics and Dentofacial Orthopedics at Damascus University between July and October 2015. Inclusion criteria were as follows: patient aged between 16 and 24 years, moderate tooth-size arch-length discrepancy of 3–5 mm with Little’s Irregularity Index (LII) of 7 mm or more in the six maxillary anterior teeth that indicates first maxillary premolars extraction, no systemic diseases, and no medications intake. 94 patients were initially examined for eligibility. 68 of those patients were excluded; 65 patients did not met the inclusion criteria and the other 3 patients declined to participate in the study due to personal conditions, resulting in 26 patients that were randomly allocated to either the laser treatment group or the placebo treatment group. No dropout occurred through the study, so the data from 26 patients were used later for the statistical analysis.

After clear explanation of the purpose and the methods of the study to the patients and/or their parents, an informed consent was obtained from each participant to confirm their approval to participate in the study.

**Fig. 1:**
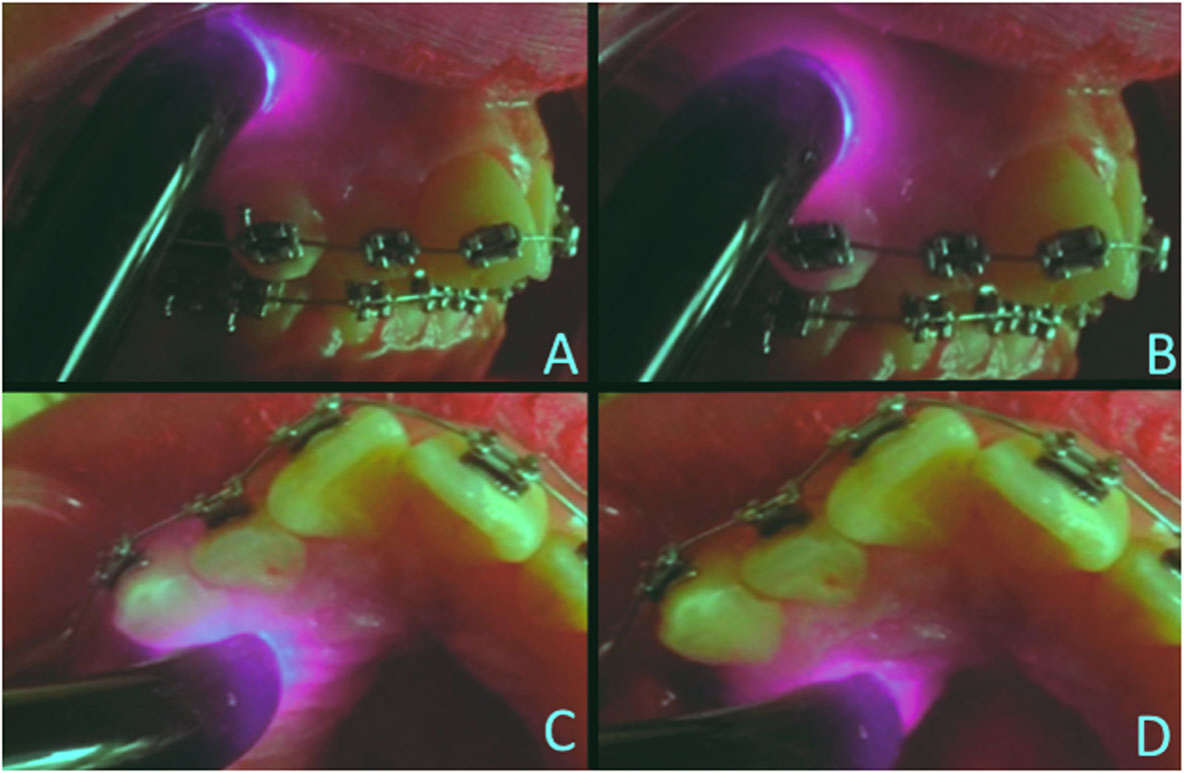
Example of LLL application points

For patients in both groups, elastomeric separators (Ortho Classic, 1300 NE Alpha Drive, McMinnville, Or, USA) were placed in the mesial and distal sides of the first maxillary molars, and the first maxillary premolars were extracted. After 7 to 10 days, molars were banded and a 0.022 in. MBT prescription fixed orthodontic appliance was bonded (American Orthodontics, Sheboygan, Wisc, USA) and a 0.014 in. NiTi archwire (American Orthodontics, Sheboygan, Wisc, USA) was inserted and engaged to all maxillary teeth using ligature wires. In the laser group, a LLL irradiation was applied immediately after initial orthodontic archwire placement to the six maxillary anterior teeth roots using a LLL device (CMS Dental ApS, 55 Wildersgade, 1408 Copenhagen K, Denmark) with a wavelength of 830 nm, an energy of 2 J per point, a power of 150 mW, and an application time of 15 s per point. LLL was applied to each root of the six maxillary anterior teeth roots. Each root was divided theoretically to apical and cervical halves. The device tip was centered in each half with direct contact perpendicular to the oral mucosa from both the buccal and palatal sides so that each tooth receives four application points (2 buccal, 2 palatal) (Fig. 1) and the total application time was 6 min. During LLLT application, both the operator and the patient wore specific protection eyeglasses to maintain eye safety. The laser device used in this study utilizes a special key called “safety key” that should be inserted in the device in order to emit the laser beam. This key is designed by the manufacturer to make sure that the eyes are protected with the eyeglasses before laser application. If the device is turned on without inserting the safety key, it will make a simple sound without emitting laser. When the key is inserted, the device makes a discrete continuous sound indicating laser emission. This property was used in this study to get the placebo effect. Since all patients would only get one treatment and were blinded about which group they were allocated to, and they would hear the device sound as if it works in both cases, so the same laser application procedure was repeated for patients in the placebo group but without inserting the safety key to apply the placebo treatment. Both treatments were applied by the first author.

The main outcome measure was the perception of pain score for both spontaneous and chewing pain at each studied time point, while the secondary outcome measures were the peak of pain and the most painful day recorded. To assess these outcome measures, patients were asked to score their pain degree at specific time points (after 1, 6, 24, 48, and 72 h of treatment application) for both spontaneous and chewing pain using a questionnaire with a 100-mm Visual Analogue Scale (VAS) for each time point (the scale starts with number 0 and ends with number 100), given that the treatment application time was unified for all patients. At each time point, patients scored their spontaneous and chewing pain degree on the VAS out of 100, with number 0 indicates no pain and number 100 indicates intolerable pain. A ruler was used to determine the exact score marked by the patient. To score their chewing pain degree, patients were instructed to chew a piece of bread with their six maxillary anterior teeth. No changes to trial outcomes occurred after the trial was commenced.

Sample size was calculated using G*power 3.1.3 program. According to Tortamano et al. study [20], the mean pain score after 3 days of LLL application was (3.9 ± 1.2) for the laser group and (5.7 ± 1.16) for the control group, which gave an effect size of 1.52. Using these data with a study power of 95%, an allocation ratio of 1:1, and a significance level of 0.05, the sample size was 13 for each group.

A simple randomization technique with allocation ratio of 1:1 (13 for each group) was implemented. Each patient was asked to pull a paper from a black box containing 26 sealed papers: 13 with the word laser and 13 with the word placebo. The patient would receive the treatment according to the picked paper. Allocation was concealed from the operator with the help of another author to prevent operator’s bias. The randomization process and participants’ assignment to interventions were also achieved by another author.

Only patients were blinded in this study. Each patient was randomly allocated to receive one treatment only so that the participant was not aware of which treatment he/she would receive. The blinding of the operator was not possible.

### Statistical analysis

Data were statistically analyzed using SPSS program version 20 (SPSS Inc., Chicago, USA). Kolmogorov-Smirnov test showed normal distribution of the data, so the independent t-test was used to compare the mean pain scores between the laser and placebo groups for both spontaneous and chewing pain at each studied time point.

## Results

**Fig. 2:**
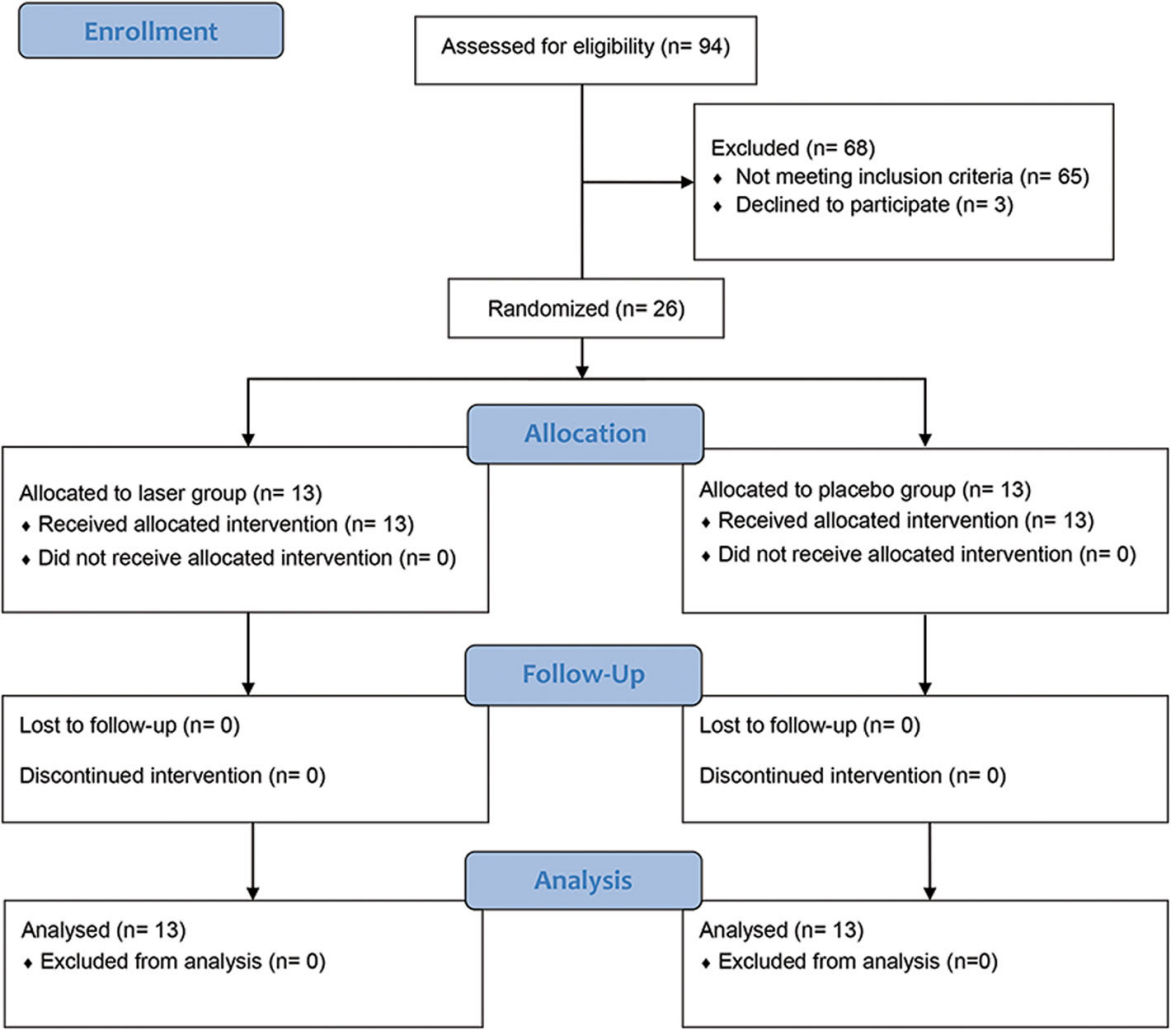
CONSORT flow diagram

Patients flow throughout the study is illustrated in Fig. 2. Table 1 shows the descriptive statistics of the sample regarding gender, age and initial LII. The total sample had an age range of 16 to 24 years [(20.07 ± 3.13) years].

**Table 1:** Sample descriptive statistics

	N	Sex		Initial LII^**a**^ (mm)		Age (years)	
		Male	Female	Mean	SD	Mean	SD
**Laser group**	13	2	11	8.91	1.57	18.53	2.9
**Placebo group**	13	4	9	10.8	2.29	21.61	2.63
**Signifigance**		0.352^b^		0.022^c^		0.009^c^	
**Total sample**	26	6	20	9.86	2.15	20.07	3.13

^a^ Little’s Irregularity Index

^b^Non-significant

^c^Significant

The pain scores of 26 patients were recorded and analyzed. The pain scores of patients in the laser group (13 patients) in all studied time points were less than their counterparts in the placebo group (13 patients) for both spontaneous and chewing pain. However, no statistically significant difference were detected between the two groups except after 72 h for chewing pain with a mean pain score for the laser group [(18.84 ± 13.44) mm] less than that for the placebo group [(38.15 ± 27.06) mm] (Tables 2 and 3).

**Table 2:** Mean pain scores of spontaneous pain (mm)

Evaluation time	Laser group		Placebo group		***P*** value
	Mean	SD	Mean	SD	
**1 Hour**	0.76	2.77	5.70	9.50	0.828^a^
**6 Hours**	3.46	6.88	11.61	13.32	0.628^a^
**24 Hours**	14.92	12.48	22	24.28	0.352^a^
**48 Hours**	10.23	9.98	15.23	21.01	0.441^a^
**72 Hours**	1.90	3.80	11.10	18.60	0.092^a^

^a^Non-significant

**Table 3:** Mean pain scores of chewing pain (mm)

Evaluation time	Laser group		Placebo group		*P* value
	Mean	SD	Mean	SD	
**1 H**	0.76	2.77	6.15	9.38	0.592^a^
**6 H**	8.30	11.44	20.92	22.14	0.080^a^
**24 H**	38.84	25.58	47.69	28.06	0.400^a^
**48 H**	30.30	20.24	48.23	27.62	0.712^a^
**72 H**	18.84	13.44	38.15	27.06	0.033^b^

^a^Non-significant

^b^ Significant

The most painful day was the first day after treatment application in both groups. The peak of pain was also recorded after 24 h of treatment application with a smaller mean pain score in the laser group compared to the placebo group for both spontaneous and chewing pain.

## Discussion

The purpose of this study was to evaluate the efficiency of LLLT in alleviating the orthodontic pain following initial orthodontic archwire placement for both spontaneous and chewing pain. It has been found that LLLT is not effective in pain reduction.

Orthodontic movements resulted from the application of orthodontic forces almost always produce a degree of pain that varies among patients. Researchers try to find an effective way to control pain without using medications. LLLT has attracted the attention as a new method with special properties that could affect orthodontic practice in many aspects like tooth movement acceleration and pain management.

Many researchers studied the effect of LLLT on pain reduction either after elastomeric separators placement or during several orthodontic treatment stages. Due to the different mechanisms of inducing pain in each stage, the results of this study will be discussed only with the results of the studies evaluated pain reduction following initial orthodontic archwire placement. In general, the results of those previous studies were in favor of emphasizing LLLT efficiency in pain reduction [8, 20, 23–25]. On the contrary, the results of this study showed no statistical significant difference in pain scores for any studied time point between the laser group and the placebo group both for spontaneous and chewing pain except after 72 h of laser application for chewing pain only (Table 4). This different result might be attributed from one side to the differences in laser application protocol and parameters, the pain evaluation method, and the inclusion criteria. On the other hand, systematic reviews stated that the design of some of the previous studies had some weaknesses and involved several degrees of bias that might affect their results [6, 12].

**Table 4:** Comparison with previous studies that evaluated LLLT application for orthodontic pain reduction following initial orthodontic archwire placement

Authors	Study design	Number of patients (M:F) and mean age (years)	intervention-group: control-group size	Active medium specifications	Energy per point (J)	Energy per tooth (J)	Energy density per point (J/cm^2^)	Application points and application time	Application frequency	Evaluation times after treatment application	Evaluation method	Effectiveness
Turhani et al. [25] (2006)	CCT	76 (30:46) 23.1	38 (LLLT):38(control)	GaAlAs 670 nm 75 mW continuous mode	2.25	2.25	4.25	One buccal point 30 s/tooth	Single application	After 6, 30, and 54 h	NRS	Yes
Tortamano et al. [20] (2009)	RCT	60 (18:42) 15.9	20 (LLLT):20 (placebo):20 (control)	GaAlAs 830 nm 30 mW continuous mode	0.5	5	5 per tooth	10 points (5 buccal - 5 palatal) 16 s/point	Single application	After 1, 2, 3, 4, 5, 6, and 7 days	NRS	Yes
Bayani et al. [8] (2016)	RCT	100 (66:34) 17.6	20 (placebo):20 (ibuprofen):20 (bite wafer):20 (low-level red laser):20 (low-level infrared laser )	GaAlAs 810 nm 200 mW continuous mode	1	6	3.6 per tooth	6 points (3 buccal - 3 lingual) 30 s/tooth	Single application	After 2 and 6 h, at bed time the same day, and after 1, 2, 3, and 7 days	VAS	Yes
Qamruddin et al. [24] (2018)	RCT (split-mouth)	42 (16:26) 19.81	42 (LLLT):42 (Placebo)	AlGaAs 940 nm 100 mW continuous mode	–	–	7	10 points (5 buccal - 5 lingual) 30 s/tooth	Single application	Consecutive 12 h intervals for 7 days	NRS	Yes
Giudice et al. [23] (2018)	RCT	84 (41:43) 16.5	30 (LLLT):30 (placebo):30 (control)	980 nm 1 W continuous mode	–	–	150 per arch	6 segments of the mandibular dental arch 150 s/arch	3 times of irradiation per application with 2 min interval	After 2, 6, and 24 h and from day 2 to day 7	NRS	Yes
This study	RCT	26 (6:20) 20.07	13 (LLLT):13 (placebo)	GaAlAs 830 nm 150 mW Continuous mode	2	8	2.25	4 points (2 buccal - 2 palatal) 15 s/point	Single application	After 1, 6, 24, 48, and 72 h	VAS	No

The statistical analysis in Table 1. showed a significant difference in initial LII values and age between the two groups. This is mainly attributed to the randomization process which ensure eliminating any recruiting bias to a definite group. For that, and regarding enrolling patients that completely match the inclusion criteria – nothing could be done regarding these results. Besides, previous studies showed that pain perception is not affected with age [27], and the LII values in both groubs is classified as severe according to the index so the differences do not have an impact on the results.

In this study, all participants aged between 16 and 24 years. Patients with little age gap and almost the same growth level were chosen to eliminate the effect of growth or bone changes on orthodontic movement and eventually on pain perception subjectivity. Beside, this helped overwhelm any differences in patients’ age between the two groups that occurred accidentally because of the random allocation procedure.

Cases with 3 to 5 mm of tooth-size arch-length discrepancy and 7 mm or more of Little’s Irregularity Index (LII) in the six maxillary anterior teeth that indicates the extraction of first maxillary premolars were chosen for patients’ enrollment in the study. It is recommended that the amount of maxillary crowding should be unified in order to get almost the same conditions for precise pain perception among patients [20], as the previous studies did not apply that. Moreover, none of them studied the LLL effect in extraction cases.

The extraction was achieved 7 to 10 days before treatment application in order to eliminate any interference with the alignment-associated pain.

A low-level laser was chosen for this study depending on previous studies’ results, which showed that its penetration into bone is more effective than the visible light. This penetration and transmission through the targeted tissues depends mainly on its wavelength that must be set in the optical window which falls between 500 and 1200 nm [20, 28]. Therefore, an 830-nm wavelength was used in this study.

One of the other important parameters is the laser beam energy. Sousa et al. suggested that the laser beam should have an energy between 1 and 2 J per point when it is used for pain reduction of the whole dental arch [18]. Another study showed the importance of using an laser energy density less than 20 J/cm^2^ to be effective because higher values may have inhibitory effect [29]. Based on those suggestions, an energy of 2 J per point with energy density of 4.25 J/cm^2^ of the laser beam were used in this study.

Laser was applied for one time only with application time of 15 s per point, so the total application time was 6 min, which made this application protocol practical if compared with others, which for example took up to 37.5 min to be achieved [20].

A Visual Analogue Scale (VAS) was used to record patients’ pain scores. Although it has some subjectivity in pain evaluation, it is considered one of the most used methods in pain evaluation studies because of its reliability in scoring pain in different time points when a big difference among participants is expected, according to systematic reviews [30].

**Fig. 3:**
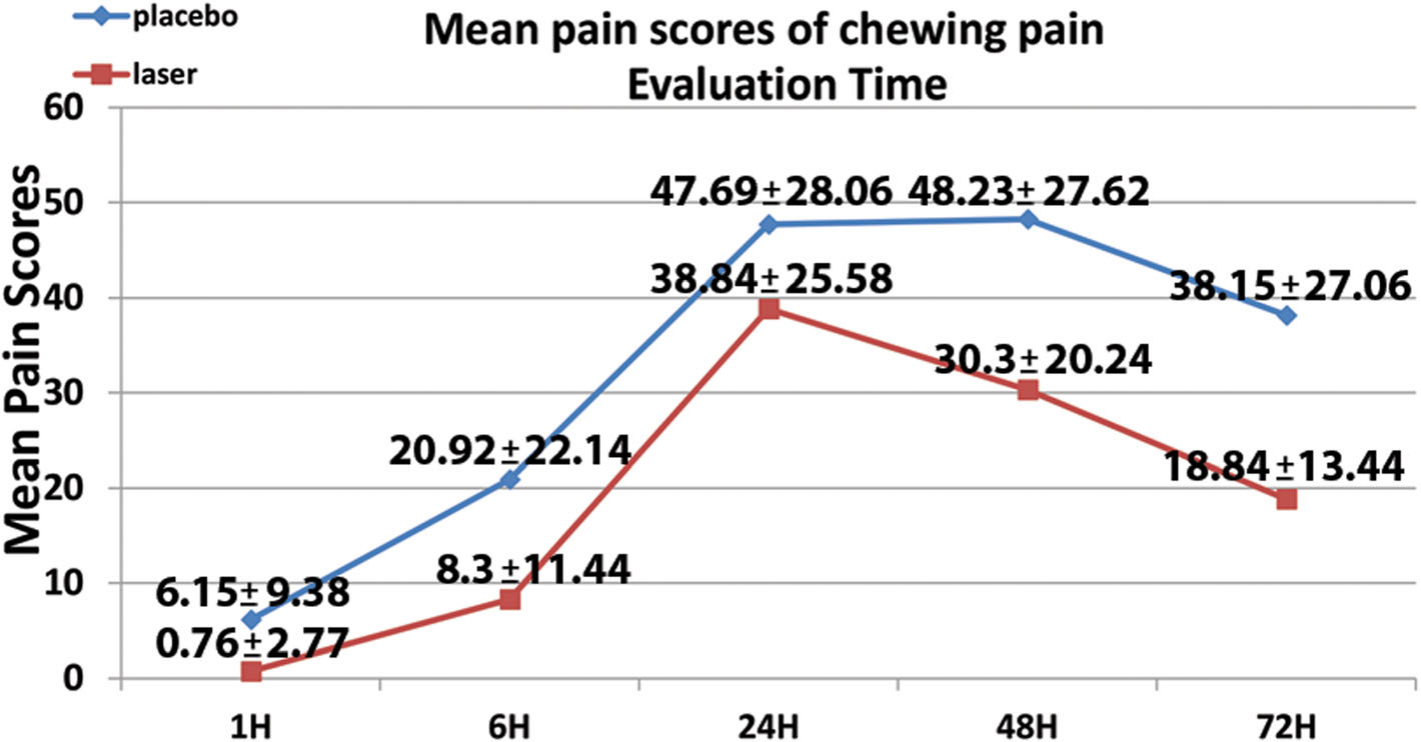
Mean pain scores of spontaneous pain

**Fig. 4:**
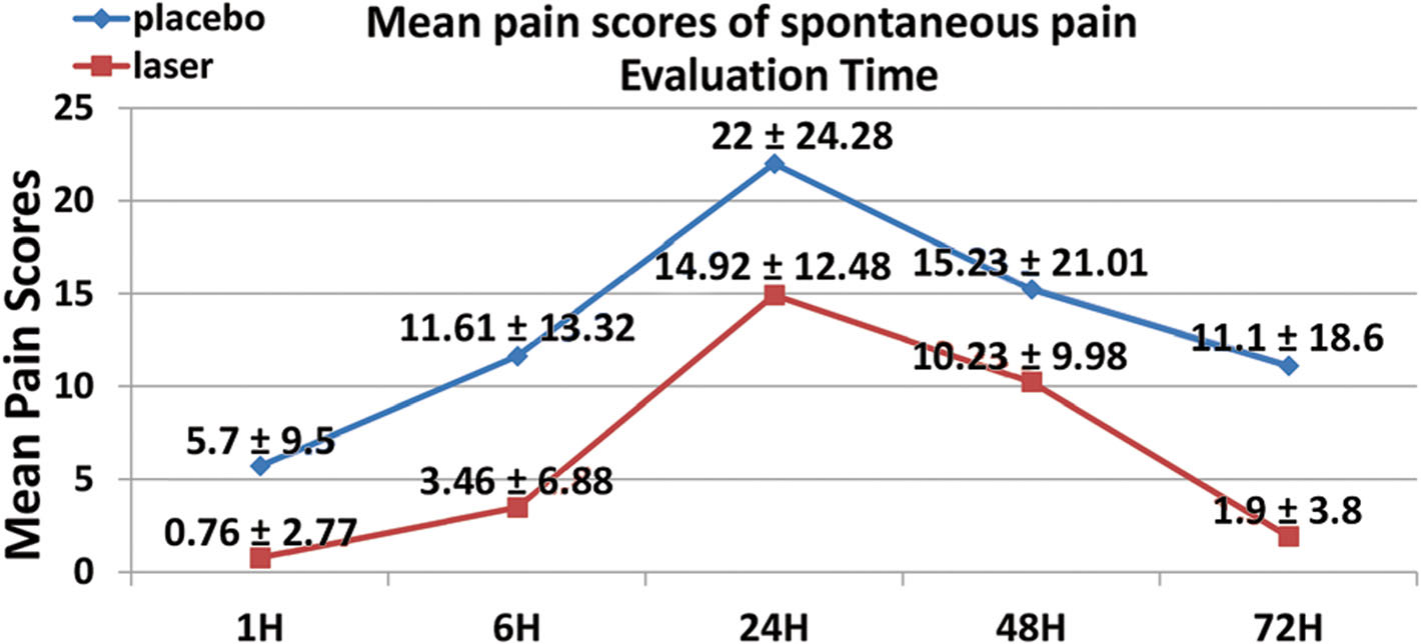
Mean pain scores of chewing pain

This study results showed that the mean pain scores of LLL treatment were less than those of the placebo treatment in all studied time points, which indicates some clinical efficiency of LLL despite the absence of statistical significance (Fig. 3 and Fig. 4). This could be explained by the large variability in the recorded pain scores, which led to big standard deviations that gave nonsignificant results.

The peak of pain was recorded after 24 h of treatment application in both groups, and then it diminishes gradually after 48 and 72 h, with less pain values scored in the laser group than the placebo group for both spontaneous and chewing pain. These results agree with the results of the previous studies, which showed that pain reaches its highest degrees after 24 h following initial orthodontic archwire placement, and then decreases starting from day 3 [3, 22].

The previous discussion explains the reasons for making our results somehow different from results and conclusions of other studies and some systematic reviews that tend to support laser efficiency in pain reduction because changing one laser parameter may lead to completely different results, indicating that LLL efficiency in pain reduction still requires further evaluation to demonstrate the role of each laser parameter in its effect on pain reduction in order to draw definite conclusions on LLL efficiency in pain reduction [12, 18, 31].

This study has some limitations. This was a single-blind trial. The operator was aware of the applied treatment, which could result in some bias risk, but applying randomization with allocation concealment helped in preventing any possible bias risk. This study applied the parallel group design, which may raise the issue of subjective differences among participants. For that, a cross-over design might be applied in future studies to avoid any possible subjectivity. On the other hand, this design prevents the carry-across effect of other designs like the split-mouth design. Besides, the sample size of this study was relatively small. Although this could be considered as a limitation that may affect the accuracy of the results, but calculating the sample size scientifically based on the results of a previous randomized controlled trial ensures that the sample size of this study is enough to have accurate and trusted results. Another limitation of this study is that it did not have a negative control group to be compared with because it would represent the normal pain status of the patients treated with fixed orthodontic appliance without any additional procedure. However, this is mainly attributed to the fact that it was enough for the primary outcome of our study which was published earlier in a separate article [19] to have only two groups, and most of the previous studies related to this topic involve only placebo and intervention group to assess the pain levels [8, 24, 25].

The general conclusion of this study is that LLL is not effective in pain reduction following initial orthodontic archwire placement. However, this result could not be generalized because laser parameters and application protocol play a major role in its effects, so different results might be obtained with future well-designed randomized controlled trials with other laser parameters and application protocols.

## Conclusions

LLLT, with the suggested parameters and under the conditions of this study, is not effective in pain reduction following initial orthodontic archwire placement.

## Data Availability

The data supporting the findings of this research can be obtained directly from the authors.

## Abbreviations

LLLT: Low-Level Laser Therapy.
LII: Little’s Irregularity Index.
VAS: Visual Analogue Scale
